# Modeling the Electron Transfer Chain in an Artificial Photosynthetic Machine

**DOI:** 10.1021/acs.jpclett.0c02766

**Published:** 2020-11-03

**Authors:** Umberto Raucci, Marika Savarese, Carlo Adamo, Ilaria Ciofini, Nadia Rega

**Affiliations:** † Dipartimento di Scienze Chimiche, Università di Napoli Federico II, Complesso Universitario di M.S. Angelo, via Cintia, I-80126 Napoli, Italy; ‡ Chimie ParisTech, PSL University, CNRS, Institute of Chemistry for Life and Health Sciences, Theoretical Chemistry and Modelling, 75005 Paris, France; § Institut Universitaire de France, 103 Boulevard Saint Michel, F-75005 Paris, France; ∥ CRIB, Centro Interdipartimentale di Ricerca sui Biomateriali P.zzale Tecchio, I-80125 Napoli, Italy

## Abstract

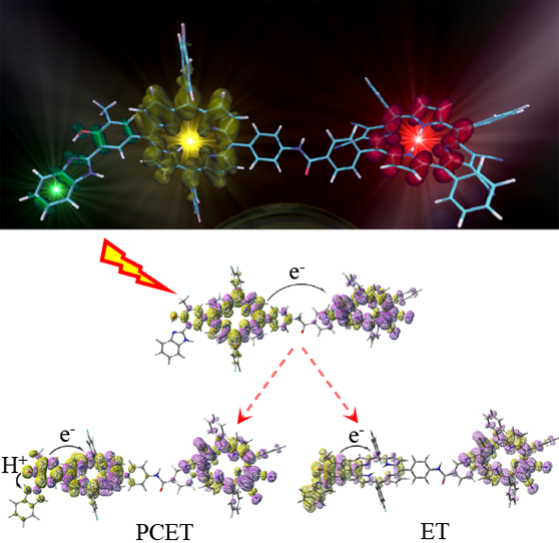

The development of efficient artificial leaves relies on the subtle combination of molecular assemblies able to absorb sunlight, converting light energy into electrochemical potential energy and finally transducing it into accessible chemical energy. The electronic design of these charge transfer molecular machines is crucial to build a complex supramolecular architecture for the light energy conversion. Here, we present an *ab initio* simulation of the whole decay pathways of a recently proposed artificial molecular reaction center. A complete structural and energetic characterization has been carried out with methods based on density functional theory, its time-dependent version, and a broken-symmetry approach. On the basis of our findings we provide a revision of the pathway only indirectly postulated from an experimental point of view, along with unprecedented and significant insights on the electronic and nuclear structure of intramolecular charge-separated states, which are fundamental for the application of this molecular assembly in photoelectrochemical cells. Importantly, we unravel the molecular driving forces of the various charge transfer steps, in particular those leading to the proton-coupled electron transfer final product, highlighting key elements for the future design strategies of such molecular assays.

Conversion of light into electric or chemical energy is undoubtedly a very attractive solution for the global energy problem.^1−10^ Analogously, storage of solar energy into chemical fuels through the development of efficient photoelectrochemical cells (PECs)^11−13^ opens the route for new and environmentally friendly energy sources. Nevertheless, the development of these devices is not straightforward because several entangled processes have to be finely combined and controlled (i.e., light harvesting, charge separation, and electron transfer). The best inspiration for the design of such devices is definitely provided by nature that developed an extremely efficient molecular machine—the photosystem II (PSII)—able to convert sunlight into chemically accessible energy.^8−10,14^

PSII uses solar photons to drive the oxidation of water to dioxygen in an amazing way, combining different specialized molecular units (e.g., chlorophyll complex P680, oxygen-evolving center, and redox-active tyrosine–histidine pair).^14−16^ Therefore, this system is an optimum starting point for the construction of artificial photosynthetic machines. In this perspective, Moore and co-workers proposed a molecular triad (hereafter named BiPhOH-PF_10_-TCNP and depicted in Figure 1) functionally mimicking the highly efficient initial photoinduced charge-separated state in PSII.^17^ This system is composed by (i) a functionalized porphyrin moiety (PF_10_) acting as primary electron donor and mimicking the chlorophyll P680 exciton trap of PSII, (ii) a tetracyanoporphyrin (TCNP) ring that acts as electron acceptor simulating the pheophytin moiety, and (iii) a benzimidazole-phenol group (BiPhOH) that models the tyrosine hystidine pair of PSII. This pair is involved in a proton-coupled electron transfer (PCET) reaction during the photosynthetic cycle. Moore and co-workers provided a detailed spectroscopic and electrochemical characterization of this molecular triad leading to the complex decay pathways reported in the inset of Figure 2.^17^ Based on experimental data, their hypothesis is that the initial excitation of the PF_10_ group is followed by singlet energy transfer to the TCNP moiety, whose excited state can relax by a photoinduced electron transfer (PET) toward a charge-separated state giving rise to a Bi-PhOH-PF_10_^**+•**^-TCNP^**–•**^ species. They proposed that this species rapidly undergoes a PCET reaction in which an electron is transferred from the phenol to the PF_10_^**+•**^ group, while the phenolic proton is transferred toward the benzimidazole group providing the final species, postulated to be the BiH^+^-PhO^**•**^-PF_10_-TCNP^**–•**^ molecule. Because time-resolved spectra suggest that this charge-separated state has a long lifetime and a high redox potential, this triad becomes particularly attractive for the development of new PEC devices.^17^

**Figure 1:**
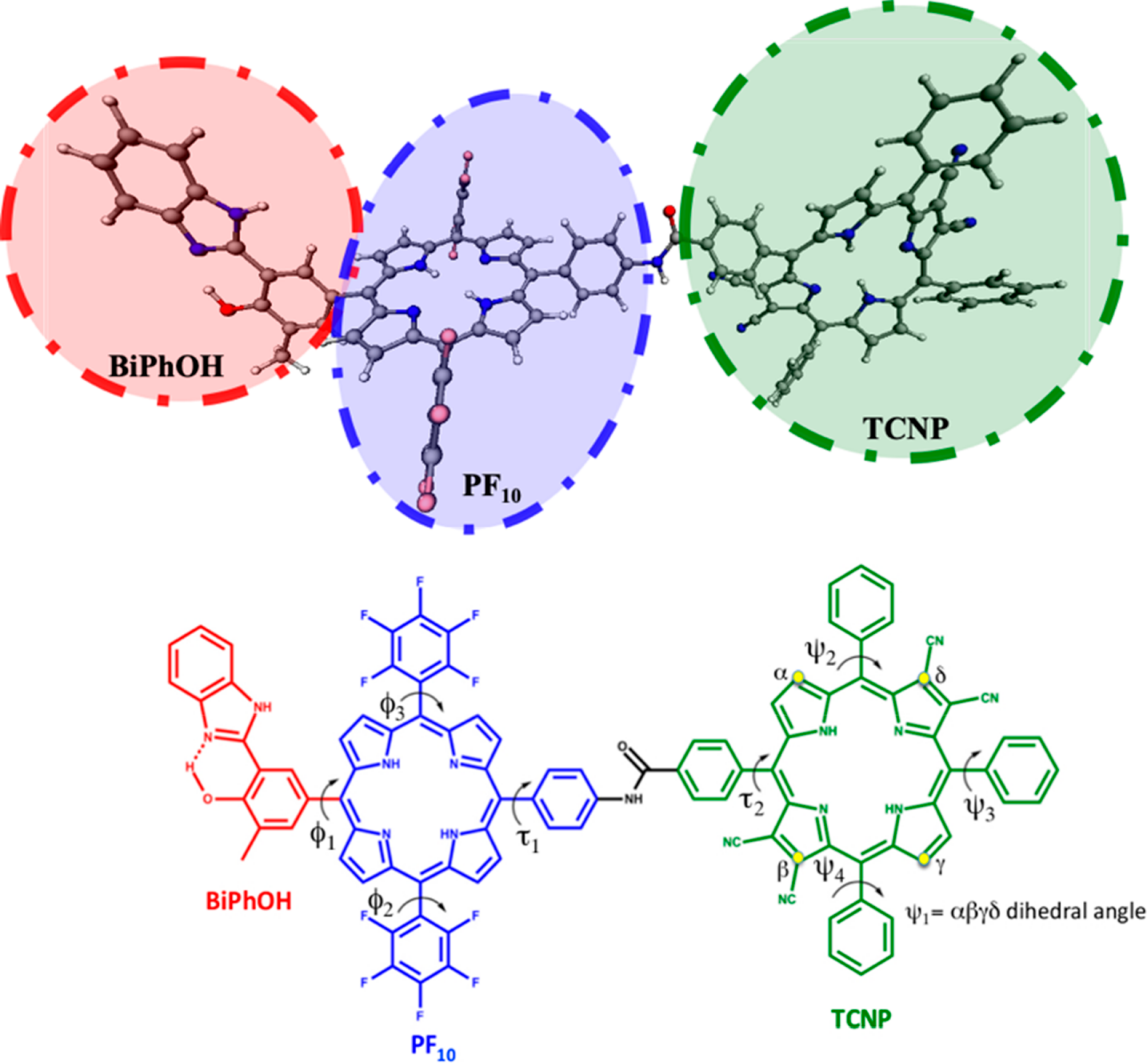
Molecular structure of triad BiPhOH-PF_10_-TCNP composed of three covalently linked subunits: BiPhOH, PF_10_, and TCNP. Labeling of critical dihedral angles is also provided. The dihedral angle Ψ_1_ is that defined by the α, β, γ, and δ atoms.

**Figure 2:**
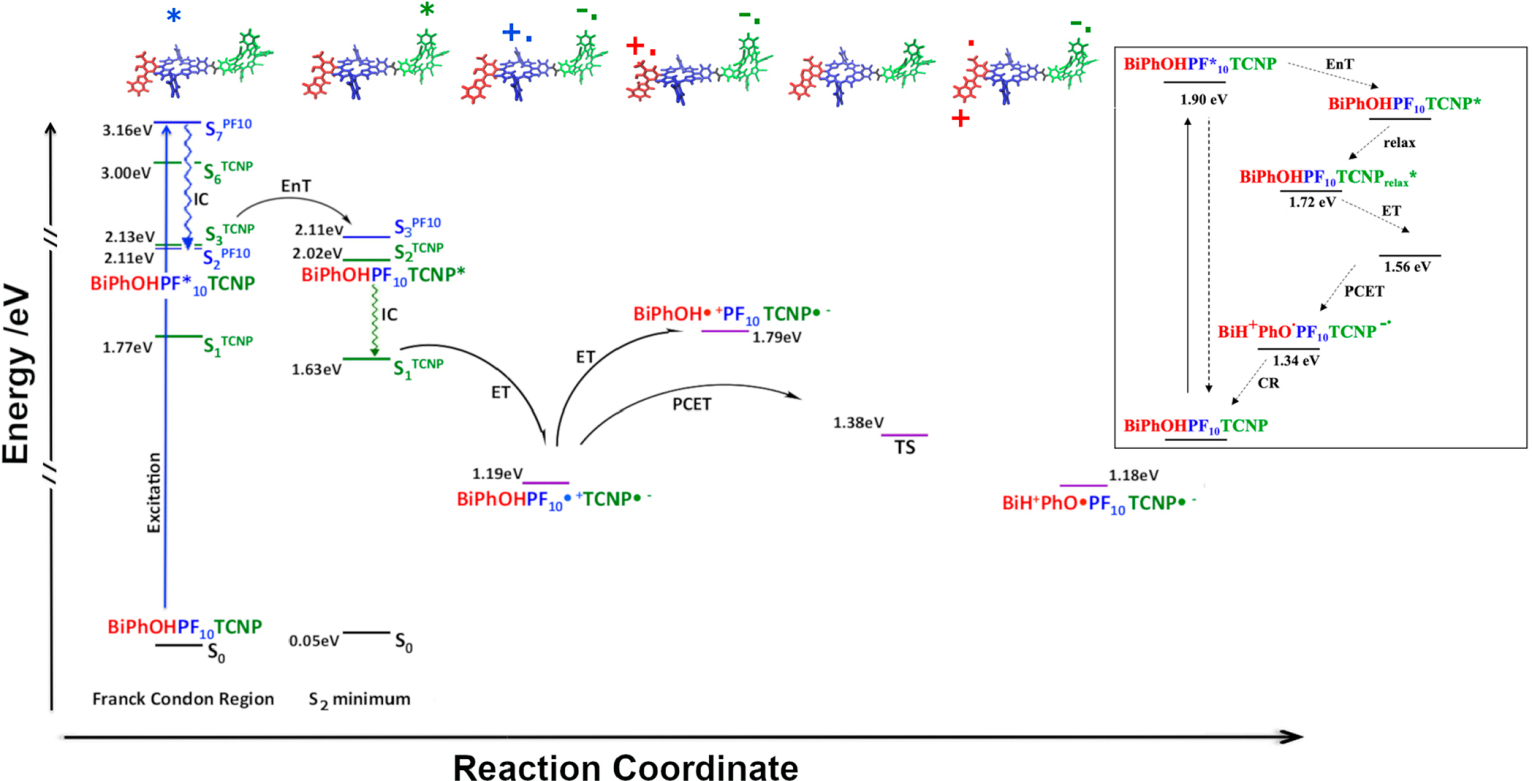
Simulated decay pathway for BiPhOH-PF_10_-TCNP. Excitation, internal conversion (IC), energy transfer (EnT), electron transfer (ET), and proton-coupled electron transfer (PCET) steps are represented. Energy levels have been computed at the TD-DFT and broken-symmetry level of theory. The vertically computed excited states are reported in the Franck–Condon region and S_2_ energy minimum. For comparison, in the inset is reported the experimentally proposed energy level diagram of decay pathways for triad according to ref (17).

Nevertheless, for the time being, the nature and the relative energies of all the electronic states involved in this complex mechanism have been only experimentally and indirectly estimated by combining redox measurements with absorption and emission data of the isolated form of the three molecular fragments composing the triad. At present, no clear direct evidence for the formation of the final PCET product has been obtained from experiments. Indeed, in a recent publication^4^ Moore and co-workers stated that “indirect evidence for PCET comes from reduction potentials of model compounds^18^ which indicate that PF_10_ would not generate sufficient driving force for the formation of the Bi-PhOH^**•+**^-PF_10_-TCNP^**–•**^ and thus implies the formation of the BiH^+^-PhO^**•**^-PF_10_-TCNP^**–•**^ state.”^4^

In order to tune and control the properties of such a triad as well as to help in the design of new systems, a more detailed knowledge of the electronic structure of all the intermediate states involved in the excited-state evolution is mandatory. Here, for the first time, we provide a complete theoretical structural and energetic characterization of all the crucial species involved in the decay pathway (Bi-PhOH-PF_10_^**+•**^-TCNP^**–•**^, Bi-PhOH^**•+**^-PF_10_-TCNP^**–•**^, and BiH^+^-PhO^**•**^-PF_10_-TCNP^**–•**^). By studying the complete triad we show how the final PCET product, BiH^+^-PhO^**•**^-PF_10_-TCNP^**–•**^, is energetically more stable than the Bi-PhOH^**•+**^-PF_10_-TCNP^**–•**^ side adduct, highlighting the principal structural differences between them. Furthermore, we investigate the proton-coupled electron transfer reaction reconstructing the reaction path and individuating the structural motifs driving it. Describing the driving force of the various charge transfer steps is one of our principal aims, especially to support the future design strategies of such molecular devices.

The theoretical simulation of the experimentally proposed decay pathways is far from being straightforward because of the necessity of accurately simulating the excited-state evolution of a large system in the presence of a medium—the solvent—which can play an important role in the stabilization of the different states.

Density functional theory (DFT)^19^ and its time-dependent counterpart (TD-DFT)^20,21^ offer valuable tools for the description of excited-state processes in condensed phase,^22−26^ although this choice makes the accurate simulation of excited states with a net intramolecular charge transfer character far from trivial.^27,28^ For this reason, we complemented the TD-DFT description of such states resorting to the broken-symmetry approach (BS) originally developed by Noodleman and co-workers^29−32^ to describe magnetic coupling^33^ and thus open shell singlet states using a single-determinant approach. This kind of approach enables the setup of computationally stable protocols for the description of an excited state dealing with charge transfer processes, which might be of interest in the design of new synthetic models for photoelectrochemical devices.

To simplify the following discussion each excited state of the triad will be labeled as S_*n*_^X^. The X superscript identifies the diabatic composition of the excited state based on the molecular moiety mostly involved in the excitation (e.g., X can be TCNP or PF_10_) while the subscript *n* specifies the adiabatic electronic state number according to its energy. S indicates that each excited state computed is a singlet. The computational details are provided in the Supporting Information.

Initially the triad is excited from its ground electronic state (S_0_). The two subunits PF_10_ and TCNP of BiPhOH-PF_10_-TCNP are almost perpendicular in S_0_, while the BiPhOH and PF_10_ rings are twisted by roughly 67° (Φ_1_ = −67°, see Figure 1 for the labeling and Table 1 for the values of the other principal dihedral angles) suggesting a certain electronic coupling among them. The PF_10_ moiety is highly symmetric with the pentafluorephenyl groups similarly oriented with respect to the plane of the porphyrin ring. The TCNP unit assumes a nonplanar structure because of steric interactions between the cyano and phenyl groups, respectively, in the beta and meso positions of the tetrapyrrolic ring. Steric interactions also lead to an orientation for the phenyl groups far from the perpendicular one (see Ψ_2_, Ψ_3_, and Ψ_4_ dihedral angles in Table 1 and Figure 1). The experimental absorption spectrum for the triad in benzonitrile shows two intense broad bands in the Soret regions centered at 2.76 and 2.92 eV, respectively. TD-DFT results show two intense electronic transitions at 3.00 eV (oscillator strength *f* = 2.966, state S_6_^TCNP^) and 3.16 eV (*f* = 2.157, state S_7_^PF10^) localized respectively on the TCNP and on the PF_10_ groups. Starting from the absorption event, the simulated pathway can be followed by inspecting Figure 2.

**Table 1:** Characteristic Dihedral Angles (in degrees, refer to Figure 1 for labels) Computed for the Triad in the Relevant Electronic States^a^

electronic state	Φ_1_	Φ_2_	Φ_3_	τ_1_	τ_2_	ψ_1_	ψ_2_	ψ_3_	ψ_4_
S_0_	–67.14	–108.88	108.38	–113.75	–113.45	–14.55	–66.54	–114.48	–67.64
S_2_^TCNP^	–68.13	–109.72	109.47	–113.84	–117.84	–18.16	–62.86	–117.68	–64.13
S_1_^TCNP^	–68.15	–109.58	109.02	–113.38	–127.11	–25.03	–52.79	–127.32	–53.64
BiPhOH-PF_10_^+•^-TCNP^–•^	–54.28	–111.88	107.24	–121.68	–117.79	–17.61	–62.92	–117.88	–64.33
BiH^+^PhO^•^-PF_10_-TCNP^–•^	–49.54	–109.92	105.38	–115.88	–118.63	–17.77	–63.03	–117.94	–64.09
BiPhOH^+•^-PF_10_-TCNP^–•^	–92.11	–109.33	108.68	–115.81	–117.99	–17.54	–63.49	–117.49	–64.21

a All parameters are computed on the relative minimum-energy structure.

After the excitation to the state S_7_^PF10^, a fast internal conversion to a state still localized on PF_10_ but at lower energy (state S_2_^PF10^) can take place (see Figure 2). This is in line with the photochemistry of porphyrin molecules that, following the absorption to higher excited electronic states, give rapid internal conversion to S_1_, from where emission is observed.^34^ The S_2_^PF10^ state has been vertically computed at 2.11 eV (*f* = 0.017) on the S_0_ minimum-energy structure and it is characterized by the transition between frontier molecular orbitals reported in Figure S1. Another excited state (S_3_^TCNP^) can be found close in energy to S_2_^PF10^, and it is calculated at 2.13 eV (*f* = 0.103) in the S_2_^PF10^ Franck–Condon region. This state is characterized by an electronic excitation completely localized on the TCNP unit.

S_2_^PF10^ and S_3_^TCNP^ are the main actors involved in the excitation transfer from the PF_10_ to the TCNP moieties. Starting from the Franck–Condon region, a change in the S_2_ locally excited character from PF_10_ to TCNP can easily occur by coupling to the S_3_ potential energy surface. In Figure S2 we report energy profiles along a linear synchronous path coordinate linking the S_2_^PF10^ and S_2_^TCNP^ energy minimum structures, clearly showing the possible change in the S_2_ character from PF_10_ to TCNP by a nonadiabatic coupling with the S_3_ potential surface.

Thus, the global energy minimum in the S_2_ adiabatic state involves an electronic excitation completely localized on the TCNP unit (S_2_^TCNP^). This step corresponds to the experimentally hypothesized energy transfer from PF_10_ to TCNP in the triad (first step in the Figure 2 inset).

The TCNP ring distortion is one the main degrees of freedom involved in the path from S_2_^PF10^ to S_2_^TCNP^. Indeed, starting from the Franck–Condon region, the Ψ_1_ dihedral angle changes by about 4° when passing to the S_2_^TCNP^ excited-state energy minimum.

The fluorescence from the TCNP is experimentally observed at 1.72 eV, and it is computed at 2.02 eV (*f* = 0.409) in the S_2_^TCNP^ excited-state energy minimum. From an experimental point of view, it has been hypothesized that this state corresponds to the reactant for the first ET process (see inset in Figure 2). Nevertheless, from our calculations it was not possible to individuate a CT character from the PF_10_ to the TCNP unit in structures close to the S_2_^TCNP^ energy minimum, which would have been a reasonable indication of the possible ET process. Indeed, in this structure another electronic excited state (S_1_^TCNP^) with energy of 1.63 eV (*f* = 0.473) has been calculated, characterized by an electronic excitation still completely localized on the TCNP group (Figure S1 for the MOs involved in the transition). The main difference between the S_2_^TCNP^ and S_1_^TCNP^ states is a significant change of both the electronic and nuclear arrangements during the S_1_^TCNP^ relaxation. As matter of fact, the charge transfer character of the S_1_^TCNP^ excitation drastically increases when going from the S_2_^TCNP^ to the S_1_^TCNP^ minimum-energy structure: while at the S_2_^TCNP^ minimum the MOs involved in the S_1_^TCNP^ transition are completely localized on the TCNP moiety, the character of charge transfer from the PF_10_ to the TCNP unit increases at the S_1_^TCNP^ minimum-energy structure (see Figure S3 for the MOs involved in the electronic excitation). Considering that the energy difference (0.39 eV) between S_1_^TCNP^ and S_2_^TCNP^ corresponds to about 3100 cm^–1^, we propose an internal conversion occurring between the S_2_^TCNP^ and S_1_^TCNP^ states and assume the S_1_^TCNP^ state as the electron-transfer reactant (Figure 2).

The distortion of the TCNP tetrapyrrolic ring is the main degree of freedom involved during the structural relaxation of S_1_^TCNP^, and it promotes the charge transfer from the PF_10_ to the TCNP unit (see Table 1). Indeed, the Ψ_1_ dihedral angle, describing the distortion of the porphyrin ring, changes from −18° to −25° during this relaxation, driving the variation in the nature of the MOs involved in the S_1_^TCNP^ electronic transition. This deformation increases the steric repulsions between the cyano and phenyl groups that, in turn, assume a more planar orientation with respect to the porphyrin ring. The TCNP deformation is, thus, a key structural motif for the charge transfer event. Interestingly, this change in the nature of the excited S_1_^TCNP^ state was observed only for calculations performed in benzonitrile solution, while it was not reproduced in the analogue S_1_^TCNP^ optimization in cyclohexane. In this case, the excitation remains localized on the TCNP moiety. This clearly indicates that only polar solvents with high dielectric constant are able to stabilize the electron transfer product, and it is in fair agreement with the experimental evidence indicating that a high quantum yield for the PET reaction is observed only in polar medium (benzonitrile). The energy of the relaxed S_1_^TCNP^ state is computed at 1.40 eV (*f* = 0.565). Within the TD-DFT framework this is the best picture of the electron transfer product. In order to describe the evolution of this charge-separated state in the ET product, the BS approach was applied. An open shell BS singlet state characterized by a spin density localized on both the PF_10_ and the TCNP groups was computed. The spin density plot and the Mulliken spin density (MSD) integrated for fragments are reported for this structure in Figure 3a, with the fragment definition provided in Figure S4.

**Figure 3:**
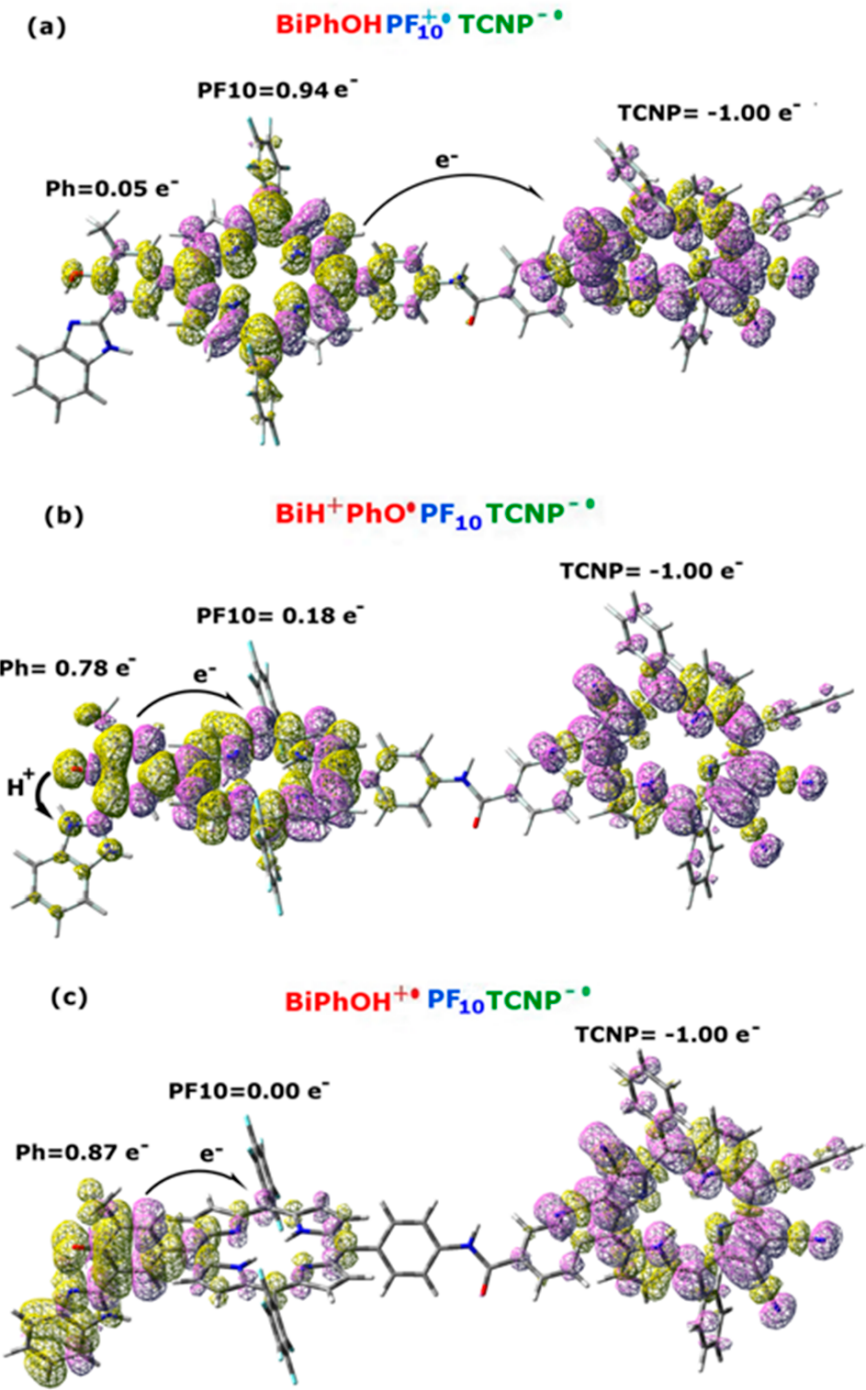
(a) Spin density plot for the electron transfer product (Bi-PhOH-PF_10_^**+•**^-TCNP ^**–•**^) in the broken-symmetry approximation. (b) Spin density for the proton-coupled electron transfer product (BiH^+^-PhO^**•**^-PF_10_-TCNP^**–•**^). (c) Spin density plot for the electron transfer product (Bi-PhOH^**•+**^-PF_10_-TCNP^**–•**^). Integration for fragments of the Mulliken spin density is also reported.

Looking at the fragments MSD it is clear that the BS-calculated singlet state represents the PET product, namely the Bi-PhOH-PF_10_^**+•**^-TCNP^**–•**^ adduct. Indeed, two unpaired electrons with opposite spin are localized on the PF_10_ and TCNP moieties, respectively, with negligible contribution on the BiPhOH group. The Mulliken charge analysis for fragments reveals a net positive charge of +1.10 on the PF_10_ unit and a net negative charge of −0.93 on the TCNP fragment. This configuration is that expected following an electron transfer from the PF_10_ to the TCNP group. The Bi-PhOH-PF_10_^**+•**^-TCNP^**–•**^ species, in turn, represents the reactant for the next PCET step.

In order to characterize the PCET reaction, we chose the phenol oxygen–hydrogen (O_Ph_H) distance as the degree of freedom representative of the proton transfer coordinate. We obtained an energy profile by a relaxed scan along this coordinate at the TD-DFT level. The S_1_^TCNP^ electronic structure showed only a negligible variation along this coordinate, which means no ET accompanied the PT event.

On the other hand, a BS solution was obtained on the PT product, corresponding to the BiH^+^-PhO^**•**^-PF_10_-TCNP^**–•**^ PCET adduct. In Figure 3b we show the spin density plot and the MSD integrated for fragments of this species. This latter analysis shows the electronic holes localized on both the TCNP (−1.0e^–^) and phenol (0.78e^–^) units. The O_Ph_H distance is 1.863 Å in this structure, while the imidazole nitrogen–hydrogen (N_Im_–H) distance is 1.023 Å.

We also observed a small spin polarization on the PF_10_ moiety. This is principally due to the strong electronic coupling between the BiPhOH and PF_10_. The transition state (TS) for the PCET step has been also computed (the spin density plot is reported in Figure S5), with an imaginary frequency at −1169 cm^–1^ (the displacement vectors for the imaginary frequency at the transition state are reported in Figure S6). The O_Ph_H and N_Im_–H distance are 1.272 and 1.205 Å, respectively, at the transition state. The integration of the intrinsic reaction coordinate has been also carried out in order to follow the variation of the spin density along the reaction path (Figure 4a). Figure 4a shows that starting from the Bi-PhOH-PF_10_^**+•**^-TCNP^**–•**^ species, an electron is transferred from the phenol toward the PF_10_ group, saturating its electronic hole when the proton is bonded to the imidazole nitrogen. The spin density on the TCNP fragment is constant, revealing the formation of the BiH^+^-PhO^**•**^-PF_10_-TCNP^**–•**^ species. In spite of a barrier of 4.44 kcal/mol, the PCET product is slightly favored by about 0.16 kcal/mol.

**Figure 4:**
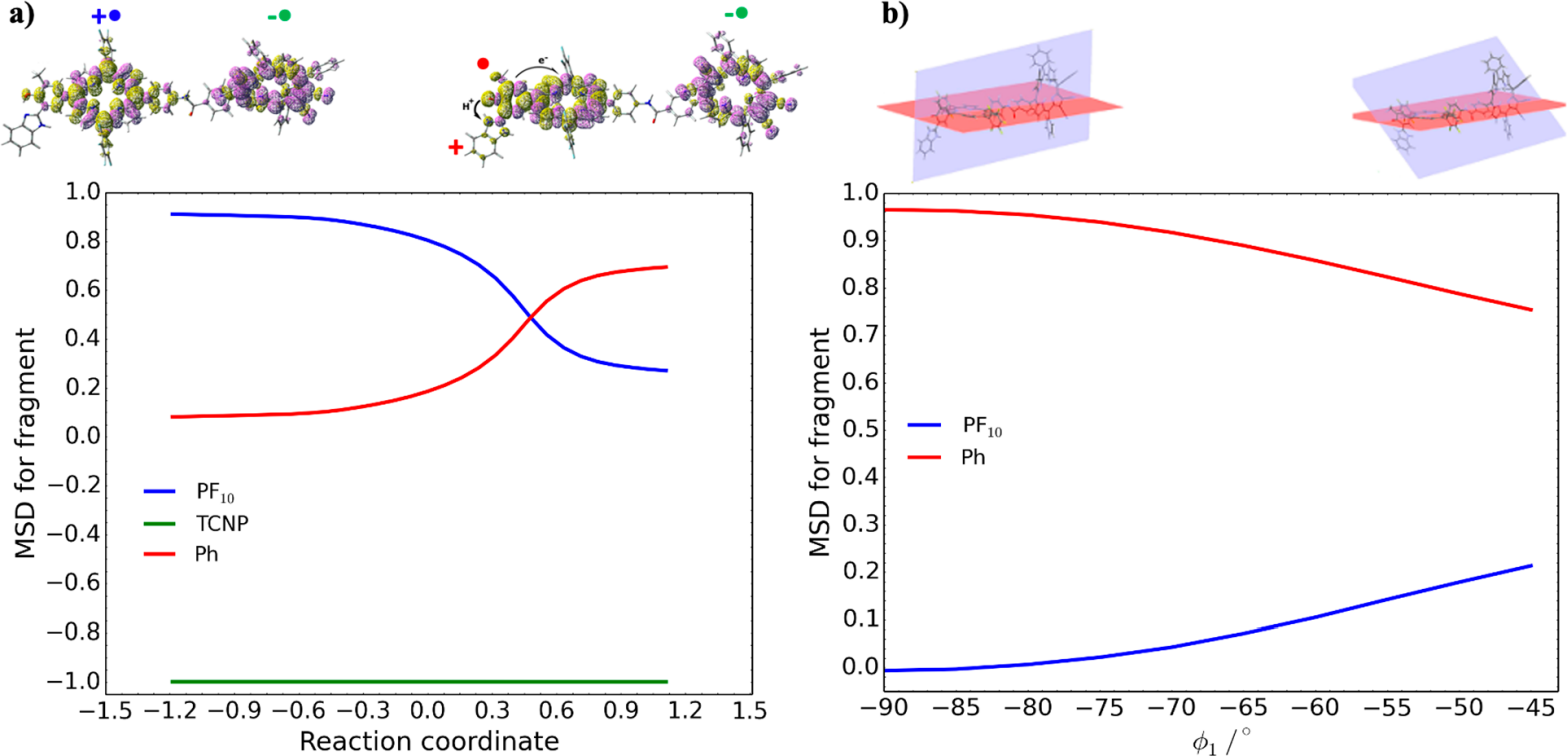
(a) Mulliken spin density (MSD) integrated for fragment along the IRC for the PCET reaction. (b) Mulliken spin density integrated for fragment along the variation of the Φ_1_ dihedral angle on the BiH^+^-PhO^**•**^-PF_10_-TCNP^**–•**^ state.

From a mechanistic point of view, the Φ_1_ dihedral angle is the degree of freedom principally involved in the PCET reaction (see Table 1). This angle defines the relative orientation between the BiPhOH and the PF_10_ units varying by about 5° during the reaction. Φ_1_ is an important parameter controlling the electronic coupling between the two units, thus modulating the electron transfer among them. To further analyze this point, the variation of the MSD on the PF_10_ and Ph fragments with respect to the Φ_1_ dihedral angle is reported in Figure 4b. This plot has been obtained scanning the Φ_1_ dihedral angle from −45° to −90° on the broken-symmetry BiH^+^-PhO^**•**^-PF_10_-TCNP^**–•**^ state. When the two rings are almost perpendicular (Φ_1_ = −90°), the spin density on the PF_10_ fragment is zero and that on Ph is about one. When the Φ_1_ angle is varied, the spin density on the PF_10_ moiety gradually increases, reaching its maximum value (about 0.2) when the two rings become more planar. At the same time the spin density on the Ph fragment decreases, reaching at least the value of about 0.8. This internal mode is crucial to drive the PCET event, and it has to be considered as a critical motif for the future design of these charge transfer molecular machines.

We also computed the alternative BS solution corresponding to the transfer of an electron from the Ph group toward the PF_10_ one, with no PT between phenol and benzimidazole groups, namely, the Bi-PhOH^**•+**^-PF_10_-TCNP^**–•**^ species (Figure 3c).

This state is found to lie 0.60 eV (13.89 kcal/mol) higher in energy with respect to the Bi-PhOH-PF_10_^**+•**^-TCNP^**–•**^ one (Figure 2). Its formation is, thus, energetically unfavorable. Once again Bi-PhOH^**•+**^-PF_10_-TCNP^**–•**^ and Bi-PhOH-PF_10_^**+•**^-TCNP^**–•**^ differ principally for the mutual orientation of the BiPh and PF_10_ moiety, namely for the Φ_1_ dihedral angle (Table 1). Indeed, this degree of freedom changes by about 40° between the two structures, with the Bi-PhOH^**•+**^-PF_10_-TCNP^**–•**^ species favored by the perpendicular arrangement between the Ph and PF_10_ rings.

In conclusion, the complete excited-state cascade of the triad BiPhOH-PF_10_-TCNP has been simulated within the TD-DFT and BS frameworks. This combined approach allowed us to describe the complexity of the structural and electronic evolution of the charge transfer steps following the electronic excitation of the triad. Furthermore, the internal degrees of freedom involved during the various steps have been successfully analyzed. We individuate the dihedral angles involved in the modulation of the electronic coupling between the BiPhOH and PF_10_ moieties as crucial parameters for the formation of the various charge transfer species.

The combination of TD-DFT and broken symmetry approaches paves the way to disentangle the complex electronic structure of PSII mimics and for the successful design of charge transfer molecular machines suitable for artificial photosynthesis.

## Supporting Information Available

Computational Details; natural transition orbitals involved in the transition leading to S_2_^PF10^, S_2_^TCNP^, and S_1_^TCNP^ excited states; evolution of S_3_^TCNP^ and S_2_^PF10^ excited states along the linear synchronous path connecting the S_2_^PF10^ and S_2_^TCNP^ energy minima; natural transition orbitals involved in the transition leading to the relaxed S_1_^TCNP^ state; definition of the molecular fragments Bi, Ph, PF_10_, and TCNP; spin density plot for the proton-coupled electron transfer transition state in the broken-symmetry approximation; displacement vectors for the imaginary frequency computed at the proton-coupled electron transfer transition state (PDF)
