# Supplementary material for: Modeling the Electron Transfer Chain in an Artificial Photosynthetic Machine
Source: J Phys Chem Lett. 2020 Nov 3;11(22):9738–44. doi: 10.1021/acs.jpclett.0c02766 (PMC8016191; doi:10.1021/acs.jpclett.0c02766)
Supplement: Supplementary file 1 — jz0c02766_si_001.pdf [file jz0c02766_si_001.pdf]

Supporting Information: Modeling the Electron Transfer Chain in an Artificial Photosynthetic Machine

Umberto Raucci[1], Marika Savarese[1], Carlo Adamo[2,3], Ilaria Ciofini[2], and Nadia Rega[1,4,*]

[1]*Dipartimento di Scienze Chimiche, Università di Napoli Federico II, Complesso Universitario di M.S. Angelo, via Cintia, I-80126 Napoli, Italy.*

[2]*Chimie ParisTech, PSL University, CNRS, Institute of Chemistry for Life and Health Sciences, Theoretical Chemistry and Modelling, 75005 Paris, France.*

[3]*Institut Universitaire de France, 103 Boulevard Saint Michel, F-75005 Paris, France.*

[4]*CRIB, Centro Interdipartimentale di Ricerca sui Biomateriali P.zzale Tecchio, I-80125 Napoli, Italy.*

**correspondence nadia.rega@unina.it*

Supplementary Information table of contents:

Computational Details

Figure S1. Natural transition orbitals involved in the transition leading to S_2^{PF10}, S_2^{TCNP} and S_1^{TCNP} excited states.

Figure S2. Evolution of S_3^{TCNP} and S_2^{PF10} excited states along the linear synchronous path connecting the S_2^{PF10} and S_2^{TCNP} energy minima.

Figure S3. Natural transition orbitals involved in the transition leading to the relaxed S_1^{TCNP} state

Figure S4. Definition of the molecular fragments Bi, Ph, PF_{10}, TCNP. They have been used to analyse the Mulliken Spin density.

Figure S5. Spin density plot for the proton coupled electron transfer transition state in the broken symmetry approximation.

Figure S6. Displacement vectors for the imaginary frequency computed at the proton coupled electron transfer transition state

Computational Details

Ground and Excited state potential energy surface (PESs) were defined at DFT[1] and TD-DFT[2-3] level, respectively. Ground state structure optimizations have been carried out at CAM-B3LYP[4] level of theory adopting the 6-31G(d,p) basis set. Excited state properties have been evaluated using CAM-B3LYP functional that gives a more reliable description of the various excited states compared to other density functionals. Amos and co-workers[5] showed that CAM-B3LYP predicts qualitatively reasonable spectra for porphyrin, in excellent agreement with complete-active-space plus second-order Moller- Plesset perturbation theory[6] and symmetry-adapted cluster configuration interaction calculations.[7] Excited state structure optimizations were performed employing 6-31G(d) basis set. Single point calculations at 6-31G(d,p) level of theory have been carried out on the excited state optimized minima to refine the excitation energy values. Solvent effects were included by the means of PCM[8-9] in its conductor-like version (CPCM),[10-11] considering benzonitrile as solvent. In the excited state the PCM linear response formalism has been adopted. Broken Symmetry solutions for Bi-PhOH-$PF_{10}^{+\cdot}$-$TCNP^{-\cdot}$, Bi-$PhOH^{\cdot+}$-PF_{10}-$TCNP^{-\cdot}$ and BiH^{+}-$PhO^{\cdot}$-PF_{10}-$TCNP^{-\cdot}$ have been obtained at CAM-B3LYP/6-31G(d,p)/CPCM level of theory. Bi-$PhOH^{\cdot+}$-PF_{10}-$TCNP^{-\cdot}$ has been optimized employing the following convergence criteria: maximum step size of 0.01 au and an RMS force of 0.0017 au. The transition state for the PCET step has been also located in the broken symmetry framework. Its nature has been confirmed by a frequency calculation showing an imaginary frequency at 1169i cm^{-1}. The integration of the intrinsic reaction coordinate for the PCET reaction has been performed by combining the first-order Euler predictor approach with a modified Bulrisch−Stoer integrator for the corrector algorithm.[12-16] All calculations were performed with the Gaussian G09 suite of programs.[17]

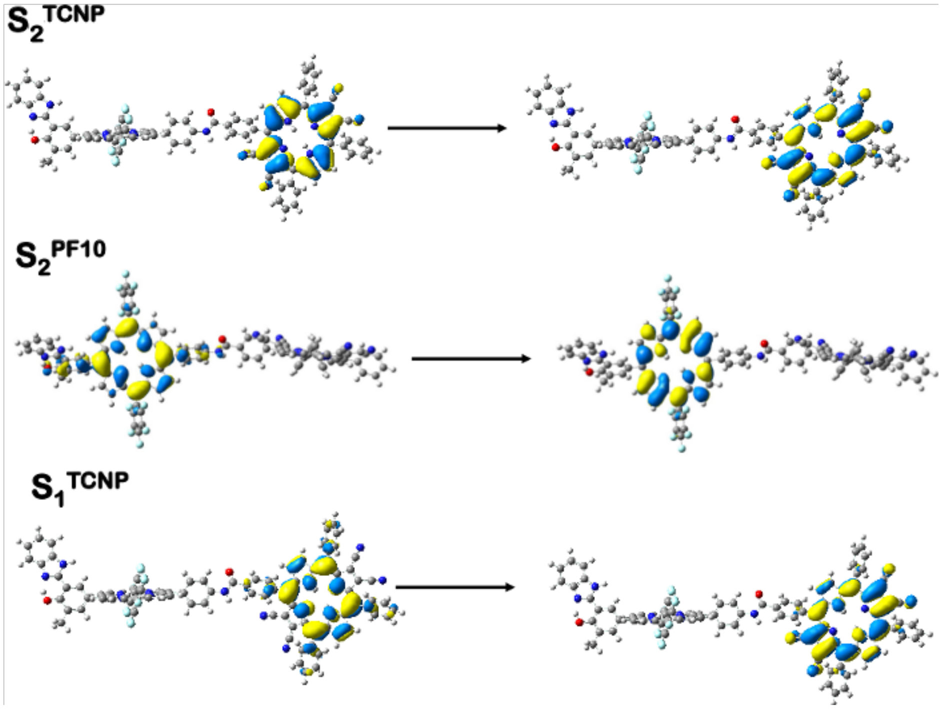

Figure S1. Natural transition orbitals involved in the transition leading to S_2^{PF10} (computed on the S_2^{PF10} state energy minimum), S_2^{TCNP} and S_1^{TCNP} (computed on the S_2^{TCNP} state energy minimum).

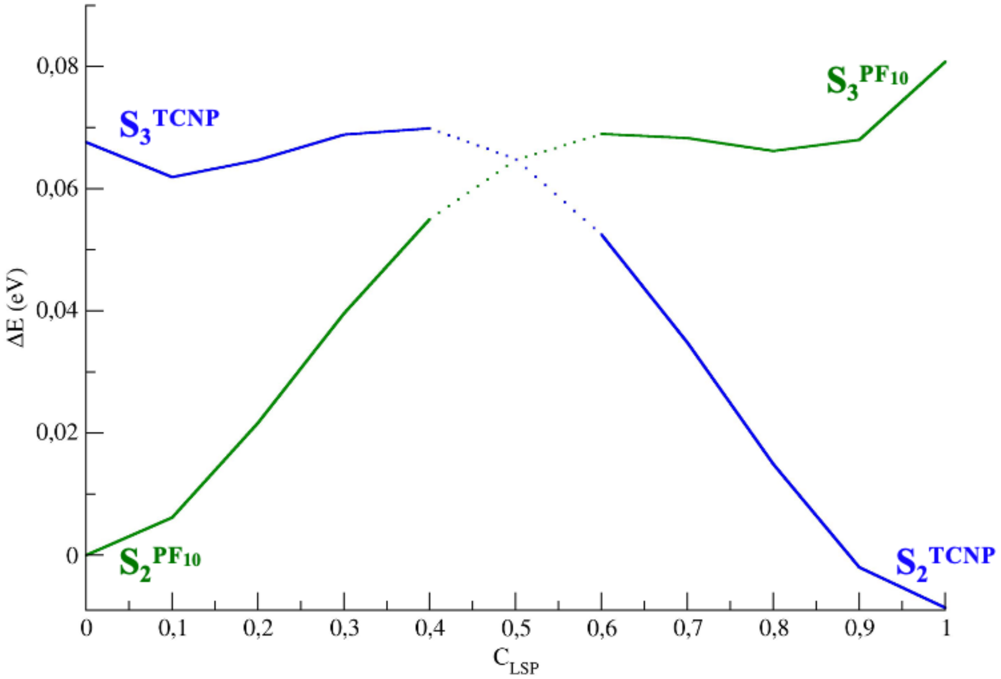

Figure S2. Evolution of S_3^{TCNP} and S_2^{PF10} excited states along the linear synchronous path connecting the S_2^{PF10} and S_2^{TCNP} energy minima.

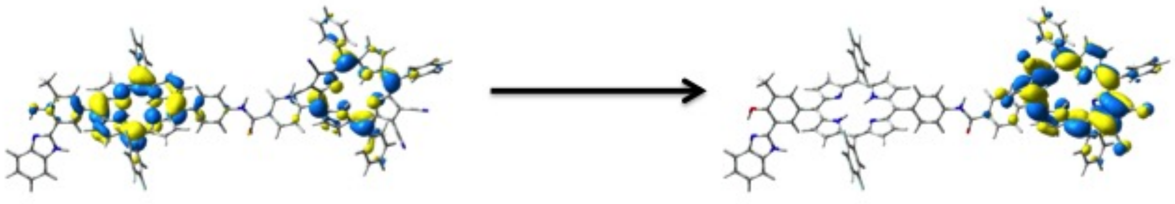

Figure S3. Natural transition orbitals involved in the transition leading to the relaxed S_1^{TCNP} state.

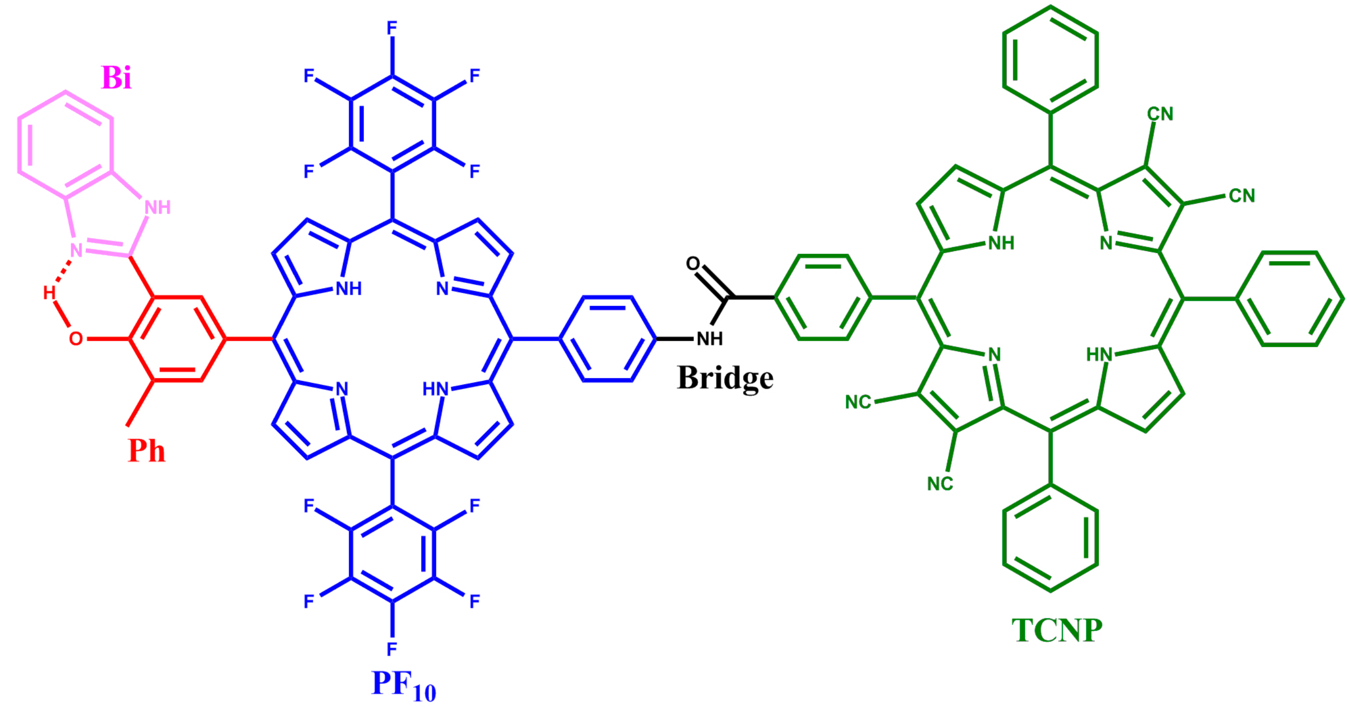

Figure S4. Definition of the molecular fragments Bi, Ph, PF10, TCNP. They have been used to analyse the Mulliken Spin density.

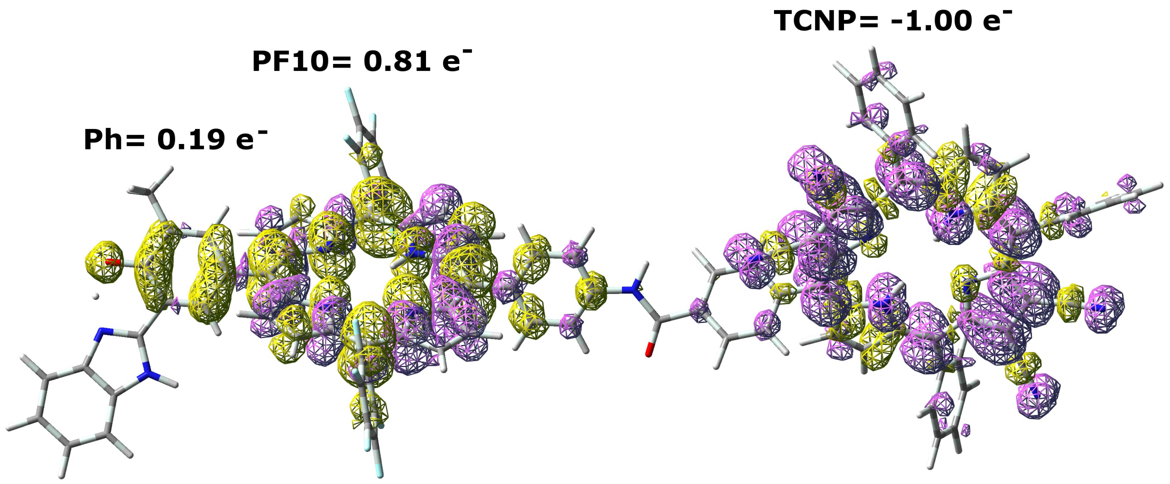

Figure S5. Spin density plot for the proton coupled electron transfer transition state in the broken symmetry approximation. Integration for fragments is also reported.

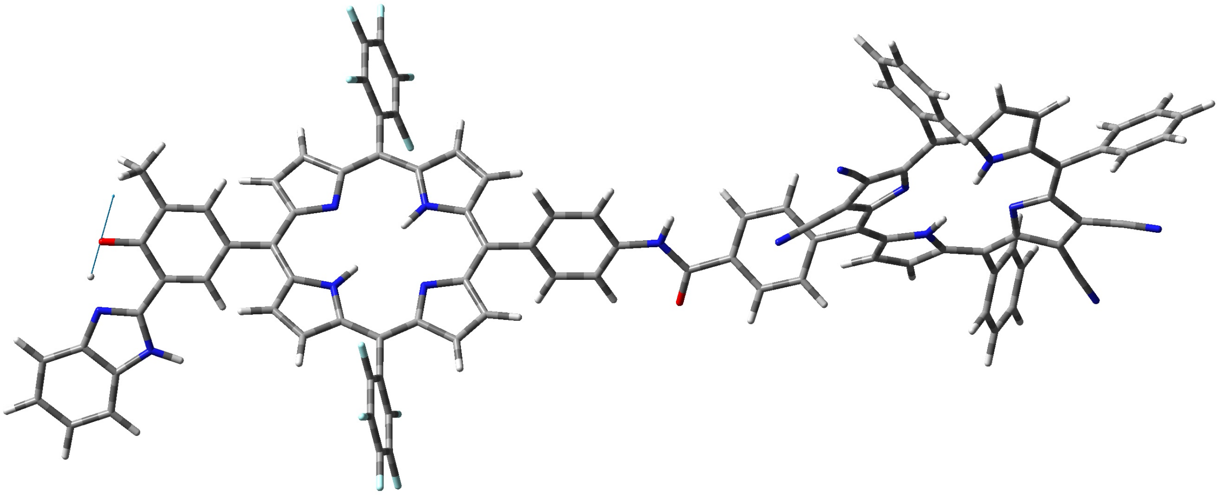

Figure S6. Displacement vectors for the imaginary frequency computed at proton coupled electron transfer transition state.

References

1. Parr, R. G. In *Density Functional Theory of Atoms and Molecules*, Horizons of Quantum Chemistry, Dordrecht, 1980//; Fukui, K.; Pullman, B., Eds. Springer Netherlands: Dordrecht, 1980; pp 5-15.
2. Runge, E.; Gross, E. K. U. Density-Functional Theory for Time-Dependent Systems. *Physical Review Letters* **1984,** *52* (12), 997-1000.
3. Stratmann, R. E.; Scuseria, G. E.; Frisch, M. J. An efficient implementation of time-dependent density-functional theory for the calculation of excitation energies of large molecules. *The Journal of Chemical Physics* **1998,** *109* (19), 8218-8224.
4. Yanai, T.; Tew, D. P.; Handy, N. C. A new hybrid exchange–correlation functional using the Coulomb-attenuating method (CAM-B3LYP). *Chemical Physics Letters* **2004,** *393* (1), 51-57.
5. Cai, Z.-L.; Crossley, M. J.; Reimers, J. R.; Kobayashi, R.; Amos, R. D. Density Functional Theory for Charge Transfer: The Nature of the N-Bands of Porphyrins and Chlorophylls Revealed through CAM-B3LYP, CASPT2, and SAC-CI Calculations. *The Journal of Physical Chemistry B* **2006,** *110* (31), 15624-15632.
6. Finley, J.; Malmqvist, P.-Å.; Roos, B. O.; Serrano-Andrés, L. The multi-state CASPT2 method. *Chemical Physics Letters* **1998,** *288* (2), 299-306.
7. Nakatsuji, H. Cluster expansion of the wavefunction. Electron correlations in ground and excited states by SAC (symmetry-adapted-cluster) and SAC CI theories. *Chemical Physics Letters* **1979,** *67* (2-3), 329-333.
8. Tomasi, J.; Mennucci, B.; Cammi, R. Quantum mechanical continuum solvation models. *Chemical reviews* **2005,** *105* (8), 2999-3094.
9. Scalmani, G.; Frisch, M. J.; Mennucci, B.; Tomasi, J.; Cammi, R.; Barone, V. Geometries and properties of excited states in the gas phase and in solution: Theory and application of a time-dependent density functional theory polarizable continuum model. *The Journal of chemical physics* **2006,** *124* (9), 094107.
10. Barone, V.; Cossi, M. Quantum calculation of molecular energies and energy gradients in solution by a conductor solvent model. *The Journal of Physical Chemistry A* **1998,** *102* (11), 1995-2001.
11. Cossi, M.; Rega, N.; Scalmani, G.; Barone, V. Energies, structures, and electronic properties of molecules in solution with the C‐PCM solvation model. *Journal of computational chemistry* **2003,** *24* (6), 669-681.
12. Fukui, K. The role of frontier orbitals in chemical reactions (Nobel Lecture). *Angewandte Chemie International Edition in English* **1982,** *21* (11), 801-809.
13. Gonzalez, C.; Schlegel, H. B. An improved algorithm for reaction path following. *The Journal of Chemical Physics* **1989,** *90* (4), 2154-2161.
14. Schlegel, H. B. Geometry optimization on potential energy surfaces. In *Modern Electronic Structure Theory: Part I*, World Scientific: 1995; pp 459-500.
15. Hratchian, H. P.; Frisch, M. J.; Schlegel, H. B. Steepest descent reaction path integration using a first-order predictor–corrector method. *The Journal of chemical physics* **2010,** *133* (22), 224101.
16. Hratchian, H. P.; Schlegel, H. B. Finding minima, transition states, and following reaction pathways on ab initio potential energy surfaces. In *Theory and applications of computational chemistry*, Elsevier: 2005; pp 195-249.

17. Frisch, M.; Trucks, G.; Schlegel, H. B.; Scuseria, G. E.; Robb, M. A.; Cheeseman, J. R.; Scalmani, G.; Barone, V.; Mennucci, B.; Petersson, G. Gaussian 09, revision D. 01. Gaussian, Inc., Wallingford CT: 2009.
